# Exendin-4 Increases Scavenger Receptor Class BI Expression via Activation of AMPK/FoxO1 in Human Vascular Endothelial Cells

**DOI:** 10.3390/cimb44110370

**Published:** 2022-11-03

**Authors:** Jingya Lyu, Hitomi Imachi, Kensaku Fukunaga, Seisuke Sato, Toshihiro Kobayashi, Takanobu Saheki, Salimah Japar, Hisakazu Iwama, Yuta Matsumura, Miyo Ozaki, Takafumi Yoshimura, Koji Murao

**Affiliations:** 1 Department of Physiology, School of Medicine, Jinan University, 601 Huangpu Avenue West, Guangzhou 510632, China; 2 Department of Endocrinology and Metabolism, Faculty of Medicine, Kagawa University, 1750-1 Ikenobe, Miki-cho 761-0793, Japan; 3 Life Science Research Center, Kagawa University, 1750-1 Ikenobe, Miki-cho 761-0793, Japan; 4 Department of Clinical Laboratory, Faculty of Medicine, Kagawa University, 1750-1 Ikenobe, Miki-cho 761-0793, Japan

**Keywords:** human SR-BI/CLA-1, activation of eNOS, HDL, GLP-1, AMPK

## Abstract

Glucagon-like peptide-1 receptor agonist (GLP-1RA) has been clinically proven to protect endothelial function. Previously, we demonstrated that endothelial NO synthase (eNOS) was activated by high-density lipoprotein (HDL) via its scavenger receptor of the B class/human homologue of SR-BI, CD36 and LIMPII analogous-1(hSR-BI/CLA-1). Here, we investigated the effect of GLP-1RA and exendin-4 on the expression of hSR-BI/CLA-1 in HUVECs. Our results confirmed that GLP-1R was expressed in HUVECs by PCR and exendin-4 significantly enhanced HDL-induced eNOS activation. Next, exendin-4 increased the expression of hSR-BI/CLA-1 and a blockade of GLP-1R cancelled this effect. Further, the hSR-BI/CLA-1 transcriptional activity was enhanced by exendin-4, which was diminished by the inhibition of AMPK or dominant-negative AMPK-α-subunit. Moreover, AMPK was phosphorylated by the activation of GLP-1R. Next, ChIP assay demonstrated that exendin-4 increased the FoxO1-binding in the hSR-BI/CLA-1 promoter by upregulation of FoxO1. Mutation of FoxO1-binding or silencing of FoxO1 cancelled the effect of exendin-4 on hSR-BI/CLA-1 expression. Exendin-4 reduced FoxO1 phosphorylation and induced its nuclear accumulation, while this effect was altered by the blocking of GLP-1R or inhibition of AMPK pathway. In summary, our results proved that exendin-4 increased hSR-BI/CLA-1 expression via the AMPK/FoxO1 pathway to activate eNOS, providing a basic mechanism underlining the protective effect of GLP-1RA on endothelial function.

## 1. Introduction

Diabetes mellitus, especially type 2 diabetes mellitus, is a global epidemic of chronic metabolic disorder, which is the ninth greatest cause of death all over the world [1]. The major complication of diabetic patients is cardiovascular disease, considered as the leading cause for mortality. The high level of blood glucose in diabetes induces vascular endothelial dysfunction and accelerates arteriosclerosis, resulting in coronary heart disease and stroke [2,3]. Glucagon-like peptide-1 receptor agonists (GLP-1 RA) were originally approved for the treatment of type 2 diabetes by enhancement of glucose-stimulated insulin secretion [4]. In vivo and in vitro experiments demonstrated that exendin-4, one of the GLP-1Ras, protects endothelial cells from apoptosis and oxidative-stress-induced autophagy, which suggested the protective effect of GLP-1RA on the function of the cardiovascular system [5,6]. The vascular endothelium can release relaxing and contractile factors, such as nitric oxide (NO), to regulate vascular permeability and cardiovascular hemostasis, and the activation of endothelial nitric oxide synthase (eNOS) to produce NO is critical to maintain normal cardiovascular function [7,8]. A previous study demonstrated that the phosphorylation of eNOS at Ser-1177 could be stimulated by the activation of the GLP-1 receptor (GLP-1R) in HUVECs to produce NO, which may be involved in improving atherosclerosis in diabetes [9,10]. Recent clinical trials on GLP-1RA have demonstrated its beneficial effect on protecting cardiovascular function, which may be associated with lower blood pressure and improvement of dyslipidemia [11,12]. However, the mechanism underlying GLP-1RA-mediated eNOS activation has not been fully investigated.

Hypercholesteremia in diabetes accelerates the pathology of atherosclerosis, and exendin-4 has been demonstrated to reduce intracellular cholesterol accumulation in diabetic endothelia cells [13]. High-density lipoprotein (HDL) mediates clearance of the cholesterol from peripheral tissue and plays an important role in reverse cholesterol transport. Numerous studies have shown that plasma HDL level is inversely related to the risk of cardiovascular disease [14,15,16]. However, not all the HDL-targeted therapies were able to reduce the cardiovascular risk [17]. Consequently, understanding the beneficial effect of HDL on endothelial cells seems to be important for exploring HDL-targeted cardiovascular therapy. Our previous studies showed that HDL could activate its receptor, the human homologue of SR-BI, CD36, and LIMPII analogous-1 (hSR-BI/CLA-1) [18]. Clinical data showed that hSR-BI/CLA-1-mediated HDL-cholesterol efflux capacity was inversely associated with clinical outcomes in coronary artery disease [19]. Moreover, hSR-BI/CLA-1 was demonstrated to be co-localized with eNOS [20], and our previous studies showed that angiotensin II suppressed the expression of hSR-BI/CLA-1 in HUVECs, which may contribute to the endothelial function regulated by the renin–angiotensin system [21]. This evidence suggests that activating the HDL receptor hSR-BI/CLA-1 is critical for protecting endothelial function. Thus, in this study we investigated the effect of GLP-1RA and exendin-4 on the regulation of hSR-BI/CLA-1 in HUVECs, which may assist understanding of the mechanism of the GLP-1RA-protective effect on cardiovascular function.

## 2. Materials and Methods

### 2.1. Cell Culture

HUVECs (passage 15–35) were originally from Dainippon Pharmaceutical Co, Ltd. and were maintained in DMEM supplemented with 10% heat-inactivated fetal bovine serum (FBS; Dainippon Pharmaceutical Co., Ltd., Tokyo, Japan), 100 U/mL penicillin and 0.1 mg/mL streptomycin. All cells were incubated in humidified 5% CO_2_ at 37 °C. When 80% confluent, the cells were starved with DMEM containing 0.5% FBS for 6 h. After starvation, the cells were treated with exendin-4 at 1, 10, and 100 nM for 24 h. To check the activation of eNOS, treated cells were incubated with HDL at 50 µg/mL for 10 min before protein harvest.

### 2.2. Western Blot

Whole-cell protein and nuclear protein were harvested using a TNE buffer or nuclear extracted reagents supplied with proteinase inhibitors. Protein was separated using SDS-PAGE and transferred to polyvinylidene difluoride membrane for immunoblotting. After blocking with 10% skim milk, the membrane was incubated with the 1st antibody for SR-BI (NB 400-104, Novus Biologicals, Littleton, CO, USA), eNOS (sc-8311, Santa Cruz Biotechnology Inc., Dallas, TX, USA), p1177-eNOS (9571; Cell Signal Technology, Danvers, MA, USA), p-AMPK (2535; Cell Signal Technology), AMPK (5832; Cell Signal Technology), p-FoxO1 (9461; Cell Signal Technology), FoxO1 (2880; Cell Signal Technology), and TFIID (Santa Cruz Biotechnology Inc, Dallas, TX, USA) at 4 °C overnight, or with GAPDH antibody (Biomol Research, Plymouth Meeting, PA, USA) at room temperature for 1 h [22]. The membrane was then incubated with the HRP-linked rabbit or mouse secondary antibody (DakoCy-tomation, Carpinteria, CA, USA) at room temperature for 1 h. Protein bands were detected by ECL (GE Healthcare; Tokyo, Japan) under the luminescent image analyzer LAS-1000 Plus.

### 2.3. Real-Time PCR

Total RNA from treated cells was isolated using RNA-Bee reagent and cDNA was synthesized using SuperScript II reverse transcriptase (Invitrogen). The expression of hSR-BI/CLA-1 was detected using the primer 5′-TTGAACTTCTGGGCAAATG-3′ and 5′TGGGGATGCCTTCAAACAC-3. Human GAPDH was used as a housekeeping gene. The real-time PCR was performed in CFX96 Touch Real Time PCR Detection Systems (BIO-RAD). The resulting values were analyzed as the relative expression compared with control levels as described previously [23].

### 2.4. Mutagenesis

To generate the plasmid with FoxO1-binding sites, mutated hSR-BI/CLA-1 promoter plasmid (pLUC-hSR-BI/CLA-1-mut), we used the hSR-BI/CLA-1 promoter plasmid (pLUC-hSR-BI/CLA-1) as the template and mutated at -769 to -766 with forward primer 5′- AGGCGGATACCTGGGAG*GGAG*GAATTGCCTGTGC-3′ and reverse primer 5′- GCACAGGCAATTCCTCC*CTCC*CAGGTATCCGCCT-3′ by PCR as instructed by the QuickChange site-directed mutagenesis kit (Agilent). The FoxO1-binding sites were mutated from AACA to GGAG, which was confirmed by DNA sequencing.

### 2.5. Transfection and Luciferase Reporter Gene Assay

AMPK-α1 subunit dominant–negative plasmid (pAMPK-α1-DN) with mutation of K45R and pLUC-hSR-BI/CLA-1 were purified and transfected into HUVECs with Lipofectamine 2000. At 24 h after transfection, HUVECs were incubated with or without various inhibitors (10 µM LY290042; 1 µg/mL STO-609; 10 µM PD98059 or 10 µM Compound C) for 30 min and then were treated with exendin-4 at 10 nM for 24 h. The luciferase activity was checked as previously described [24]. 

For the siRNA transfection, siPORT lipids (Ambion) were used to introduce the silencing of FoxO1 as previously described [25]. The siRNA-targeted sequences were shown as follows: scrambled siRNA, 5’-GGCUUAUUGUUCUUAGUAAGA-3’; and FoxO1 siRNA, 5’-GGAGAUACCUUGGAUUUUAUU-3’.

### 2.6. Chromatin Immunoprecipitation (ChIP) Assay

The ChIP-IT^TM^ kit from Active Motif was used to detect the binding of FoxO1 to the hSR-BI/CLA-1 promoter region in HUVECs. Sonicated chromatin was immunoprecipitated with 2 µg of FoxO1 antibody or negative IgG overnight. The purified DNA of each group was analyzed by PCR to harvest the region containing the putative FoxO1 response sequence (FRS) in the promoter region of hSR-BI/CLA-1. The forward primer was 5′-AGGATAGGGCCAGGCGGATAC and the reverse primer was 5′-CCCTCATCTCCGCAGTCCATC. The PCR product was 129 bp.

## 3. Results

### 3.1. Exendin-4 Induces the Phosphorylation of eNOS in HUVECs

**Figure 1:**
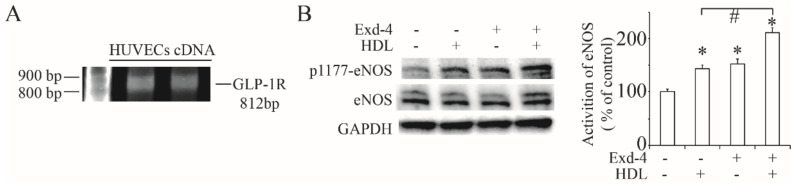
**Activation of eNOS by exendin-4 in HUVECs**. (**A**) Full-length GLP-1 receptor transcript expression as analysed by RT-PCR in HUVECs. (**B**) Phosphorylation of eNOS at Ser1177 stie stimulated by the treatment of exendin-4 at 10 nM for 24 h, pre-treating with or without HDL at 50 ng/mL for 10 min. The ratio of p1177-eNOS to eNOS is shown as percent of control. A graph showing the mean ± SEM (n = 3) of separate experiments for each treatment group is included. * *p* < 0.05 compared with control; # *p* < 0.05 compared with HDL treatment.

Firstly, we successfully cloned the GLP-1 receptor full length DNA using the cDNA from HUVECs (Figure 1A) to confirm that there was a GLP-1 receptor expressed in HUVEC cells.

In terms of the beneficial effect on cardiovascular function, we checked the effect of exendin-4 on eNOS activation and, as shown in Figure 1B, treatment of exendin-4 significantly induced the phosphorylation of eNOS at Ser1177. Moreover, the activation of eNOS was much higher with the addition of HDL, suggesting that the HDL receptor hSR-BI/CLA-1 may be involved in exendin-4-protecting cardiovascular function.

### 3.2. Exendin-4 Increases the Expression of hSR-BI/CLA-1 via GLP-1 Receptor in HUVECs

**Figure 2:**
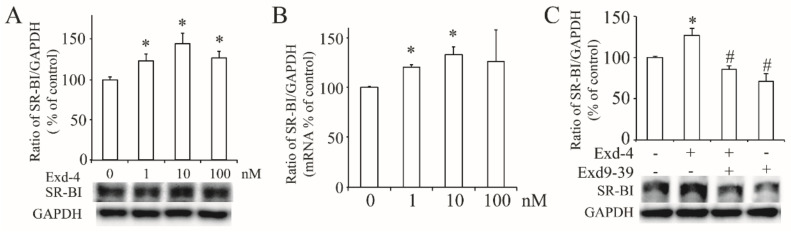
**Exendin-4 increased the expression of hSR-BI/CLA-1 via GLP-1 receptor in HUVECs.** (**A**) The protein expression of hSR-BI/CLA-1 in HUVECs treated with exendin-4 at varying doses (0~100 nM) for 24 h. (**B**) The mRNA level of hSR-BI/CLA-1 in HUVECs treated with exendin-4 at varying doses (0~100 nM) for 24 h. (**C**) The protein expression of hSR-BI/CLA-1 in HUVECs treated with exendin-4 at 10 nM together with or without exnendin9-39 for 24 h. A graph showing the mean ± SEM (n = 3) of separate experiments for each treatment group is included. The ratio of hSR-BI/CLA-1 to GAPDH is shown as percent of control. * *p* < 0.05 compared with control; # *p* < 0.05 compared with exendin-4.

Next, we checked the effect of exendin-4 on the expression of hSR-BI/CLA-1 in HUVECs, and the protein and mRNA level of hSR-BI/CLA-1 (Figure 2A,B) was dose-dependently increased, with 10 nM of exendin-4 as the optimal concentration. To verify if the GLP-1 receptor was involved in this process, we used exnedin9-39 to block the GLP-1 receptor, and the result showed that exendin-4 failed to induce the protein expression of hSR-BI/CLA-1 (Figure 2C), confirming that activation of the GLP-1 receptor is required for upregulation of SR-BI in HUVECs.

### 3.3. Exendin-4 Enhances the Promoter Activity of hSR-BI/CLA-1 via AMPK Pathway

**Figure 3:**
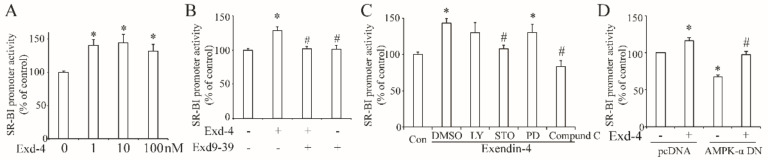
**Exendin-4 enhanced the transcriptional activity of hSR-BI/CLA-1 via AMPK signaling pathway in HUVECs**. (**A**) The promoter activity of hSR-BI/CLA-1 induced by varying doses of exendin-4 (0~100 nM) for 24 h. (**B**) The promoter activity of hSR-BI/CLA-1 in HUVECs with the treatment of exendin-4 or exendin9-39 for 24 h. (**C**) The effect of LY294002, STO-609, PD98059 or Compound C on the promoter activity of hSR-BI/CLA-1 induced by exendin-4. (**D**) The effect of dominant–negative AMPK catalytic α1 subunit on the promoter activity of hSR-BI/CLA-1 induced by exendin-4. * *p* < 0.05 compared with control; # *p* < 0.05 compared with exendin-4.

As exendin-4 significantly increases the protein and mRNA level of hSR-BI/CLA-1 in HUVECs, we proposed that exendin-4 may regulate the transcriptional activity of hSR-BI/CLA-1. Therefore, we employed the luciferase reporter plasmid, encoding the promoter region (~2000 bp upstream) of hSR-BI/CLA-1, and treatment with exendin-4 enhanced luciferase activity (Figure 3A), while this effect was diminished by the blockade of the GLP-1 receptor with exendin9-39 (Figure 3B).

Next, we tried to determine if the signaling pathway involved in exendin-4 regulated the transcription of hSR-BI/CLA-1. Thus, the specific inhibitors LY294002, STO-609, PD98059, or Compound C were used to block the PI3K, CaMKK, MEK or AMPK signaling pathway separately. As shown in Figure 3C, inhibition of CaMKK or AMPK by STO-609 or Compound C remarkably reduced the luciferase activity of hSR-BI/CLA-1 promoter (Figure 3C), suggesting that CaMKK and AMPK may regulate the effect of exendin-4 on the transcription of hSR-BI/CLA-1. As a previous study demonstrated that CaMKK is an upstream of AMPK, exendin-4 may regulate the hSR-BI/CLA-1 transcription with AMPK as a downstream. To further confirm our hypothesis, we co-transfected the plasmid containing the dominant–negative AMPK catalytic α1 subunit (AMPK-α1-DN) into HUVECs, and the dominant–negative AMPK-α1 plasmid reduced the promoter activity of hSR-BI/CLA-1 enhanced by exendin-4 (Figure 3D).

### 3.4. Exendin-4 Activates the AMPK via GLP-1 Receptor in HUVECs

**Figure 4:**
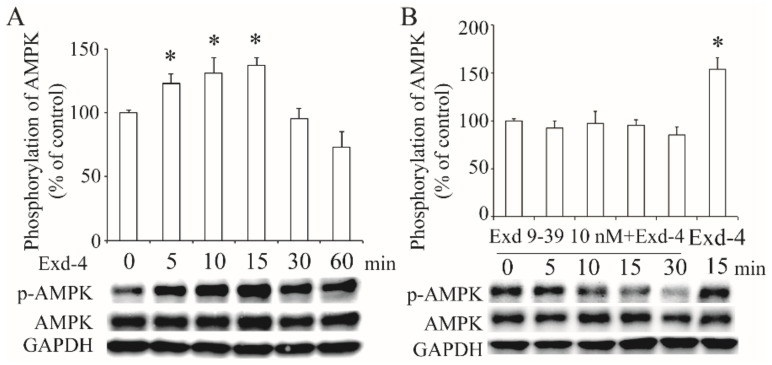
**Activation of AMPK signaling by exendin-4 via GLP-1 receptor**. The phosphorylation of the AMPK α subunit at Thr172 in HUVECs treated with exendin-4 (**A**), or together with exendin9-39 (**B**). The ratio of p-AMPK to AMPK is shown as percent of control. A graph showing the mean ± SEM (n = 3) of separate experiments for each treatment group is included. * *p* < 0.05 compared with control.

To verify the effect of exendin-4 on the activation of the AMPK signaling pathway, we treated the HUVECs with 10 nM of exendin-4 for varying amounts of time (0~60 min). As shown in Figure 4A, exendin-4 activated the phosphorylation of the AMPK α subunit at the Thr172 site. However, this activation was diminished by the blockade of the GLP-1 receptor with exneind9-39 (Figure 4B), demonstrating that exendin-4 activates AMPK signaling via the GLP-1 receptor.

### 3.5. Exendin-4 Enhances the Transcription of hSR-BI/CLA-1 via FoxO1

**Figure 5:**
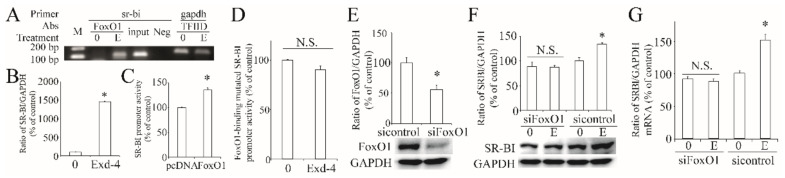
**Roles of FoxO1 in exendin-4-regulated hSR-BI/CLA-1 transcription.** (**A**) Binding of FoxO1 to the hSR-BI/CLA-1 promoter in HUVECs treated with or without exendin-4. FoxO1 antibody specifically immunoprecipitates hSR-BI/CLA-1chromatin from HUVECs by ChIP assay. No ChIP was detected when the chromatin was immunoprecipitated with unspecific negative control IgGs (Neg). Input was a positive control. (**B**) The qPCR result with the template from ChIP. (**C**) The effect of FoxO1 on hSR-BI/CLA-1 promoter activity. (**D**) The effect of exendin-4 on the promoter activity of FoxO1-binding site-mutated hSR-BI/CLA-1 promoter by altering two base pairs (GATAGT to AATAGG) in the hSR-BI/CLA-1 promoter. (**E**) siRNA specific to FoxO1 effectively decreased its protein expression. (**F**,**G**) The expression of hSR-BI/CLA-1 protein (**F**) and mRNA (**G**) levels in siFoxO1-transfected HUVECs cells treated with exendin-4. 0, sample from HUVECs without treatment; (**E**), sample from HUVECs treated with 10 nM of exendin-4 for 24 h. * *p* < 0.05 compared with control or pcDNA. N.S., no significance.

As exendin-4 regulates the transcription of hSR-BI/CLA-1, we then searched the transcription factor, which is downstream of the AMPK signaling pathway. As predicted, the hSR-BI/CLA-1 promoter region contains the putative FoxO1 response sequence (FRS). Thus, we used ChIP to confirm that FoxO1 could directly bind to the hSR-BI/CLA-1 promoter (Figure 5A) and ChIP-qPCR showed that this binding was significantly increased by treatment with exendin-4 (Figure 5B). Further, overexpression of FoxO1 significantly induced the promoter activity of hSR-BI/CLA-1 (Figure 5C). When we mutated the binding site of FoxO1 in the hSR-BI/CLA-1 promoter region, the effect of exendin-4 on the enhancement of hSR-BI/CLA-1 promoter activity did not persist (Figure 5D), suggesting that exendin-4 regulates the transcriptional activity of hSR-BI/CLA-1 via FoxO1. Next, we silenced the expression of FoxO1 via specific siRNA (Figure 5E), and exendin-4 could not regulate the protein and mRNA expression of hSR-BI/CLA-1 (Figure 5F,G), demonstrating the important role of FoxO1 in exendin-4-regulated hSR-BI/CLA-1 expression.

### 3.6. Exendin-4 Regulates the Expression of FoxO1 via AMPK Pathway

**Figure 6:**
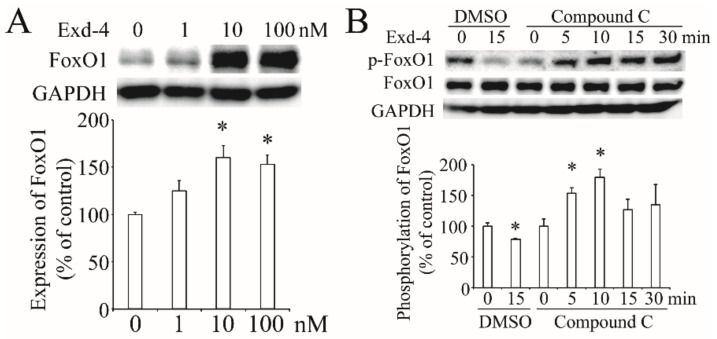
**The effect of exendin-4 on FoxO1 in HUVECs**. (**A**) Exendin-4 increased the expression of FoxO1. The ratio of FoxO1 to GAPDH is shown as percent of control. (**B**) The activation of FoxO1 at the Ser256 site stimulated by exendin-4 with or without Compound C. The ratio of p-FoxO1 to FoxO1 is shown as percent of control. A graph showing the mean ± SEM (n = 3) of separate experiments for each treatment group is included. * *p* < 0.05 compared with control.

Next, we checked the effect of exendin-4 on the expression of FoxO1 and it dose-dependently increased FoxO1 expression (Figure 6A). Further, we found exendin-4 reduced the phosphorylation of FoxO1 at the Ser256 site, while inhibition of the AMPK pathway with Compound C induced FoxO1 phosphorylation (Figure 6B). These results suggest that exendin-4 regulates FoxO1 via the AMPK signaling pathway.

## 4. Discussion

Based on the protective effect of GLP-1RA on the cardiovascular system in type 2 diabetics and our previous study showing that HDL could activate eNOS to produce NO using its receptor hSR-BI/CLA-1 in HUVECs, here we proved that GPL-1RA and exendin-4 increased the expression of hSR-BI/CLA-1 via the AMPK/FoxO1 pathway and enhanced the activation of eNOS in HUVECs.

GLP-1RA has been used for treating type 2 diabetes for years and shows its dramatic protective effect on preventing cardiovascular dysfunction in clinical trials. Thus, GLP-1RA is recommended by the American Diabetes Association as a second-line agent for treating diabetes with cardiovascular dysfunction [26]. The protective effect of GLP-1RA can be explained with three points: reducing blood pressure, improving atherogenic dyslipidemia and protecting endothelial function [11,12]. Production of NO through the activation of eNOS regulates vascular permeability and cardiovascular hemostasis, and is critical to reducing blood pressure [7,8]. In the present study, we confirmed that exendin-4 could activate eNOS at the Ser1177 site in HUVECs, which may contribute to NO generation and reduction of blood pressure. This finding is consistent with previous studies showing that GLP-1RA protects cardiovascular function via the activation of eNOS [9,10]. Moreover, we confirmed that GLP-1R is expressed in HUVECs by PCR, suggesting that GLP-1RA may protect endothelial function directly via its receptor.

Excepting the direct activation of GLP-1R by GLP-1RA, another mechanism of the activation of NO-independent GLP-1R is also suggested by a meta-analysis [27]. At the same time, long-term exendin-4 treatment was proved to improve atherogenic dyslipidemia, such as through emolliating the plasma HDL level and the reduction of total cholesterol and triglycerides in type 2 diabetic patients [28,29]. Our previous study demonstrated that HDL could activate eNOS via its receptor hSR-BI/CLA-1 [18]. This evidence suggests that GLP-1RA may protect endothelial function through the regulation of hSR-BI/CLA-1 in HUVECs. As predicted, our results showed that exendin-4 enhanced the HDL-stimulated eNOS activation and increased the protein and mRNA expression of hSR-BI/CLA-1 in a dose-dependent manner, which was cancelled by the blockade of GLP-1R with the treatment of exendin9-39. Further, the transcriptional activity of the hSR-BI/CLA-1 promoter was also enhanced by exendin-4, suggesting that the activation of GLP-1R is critical for the regulation of hSR-BI/CLA-1 transcription and expression.

One goal of our present study is to investigate the signaling pathway involved in the regulation of hSR-BI/CLA-1. Thus, we used several specific inhibitors, LY294002, STO-609, PD98059, or Compound C, to separately block the PI3K, CaMKK, MEK or AMPK signaling pathways, and we found that inhibition of the CaMKK or AMPK pathway using STO-609 or Compound C cancelled the effect of exendin-4 on the enhancement of hSR-BI/CLA-1 promoter activity, suggesting that both the CaMKK and AMPK pathways were involved in GLP-1R-regulated transcription of hSR-BI/CLA-1. Since numerous studies have proven that CaMKK serves as an upstream of AMPK to stimulate its phosphorylation at the Thr172 site, we believe that AMPK is a downstream of CaMKK in our present study. Overexpression of the dominant–negative AMPK-α subunit, the main catalytic α subunit of AMPK, diminished the effect of exendin-4 on hSR-BI/CLA-1 promoter activity. Further, phosphorylation of AMPK at the Thr172 site was activated by exendin-4, which disappeared after the blockade of GLP-1R by exendin9-39. These results indicated the important role of AMPK in exendin-4-upregulated hSR-BI/CLA-1 transcription. Previous studies demonstrated that the activation of the AMPK pathway by exendin-4 was also involved in the process of attenuating cardiac hypertrophy [30], protecting against hyperglycemia-induced cardiomyocyte pyrotosis [31] and protecting endothelial cells from lipoapoptosis [32], which may also contribute to the effect of exendin-4 on protecting cardiovascular function.

As activation of GLP-1R enhanced the transcription of hSR-BI/CLA-1 via the AMPK pathway, we then searched for the possible transcription factor that regulates hSR-BI/CLA-1, which is downstream of the AMPK pathway. According to the gene analysis results, the hSR-BI/CLA-1 promoter region contains a FoxO1 response sequence (FRS), and the ChIP result confirmed this binding. Overexpression of FoxO1 significantly increased hSR-BI/CLA-1 promoter activity, which is consistent with our previous study [21]. These results indicated that FoxO1 is a transcription factor of hSR-BI/CLA-1. Moreover, the ChIP-qPCR result showed that treatment with exendin-4 enhanced FoxO1-binding in the hSR-BI/CLA-1 promoter. However, mutation of FRS in the hSR-BI/CLA-1 promoter region or silencing of FoxO1 altered the effect of exendin-4 on the upregulation of hSR-BI/CLA-1. Following this, we demonstrated that exendin-4 could increase FoxO1 expression and induce the dephosphorylation of FoxO1, further confirming that FoxO1 is critical for the process of exendin-4-induced hSR-BI/CLA-1 expression. Several previous pieces of research have demonstrated that FoxO1 serves as a downstream of the AMPK pathway and regulates gene transcription as a transcription factor [33,34,35]. In the present study, we found that inhibition of AMPK with Compound C altered the effect of exendin-4 on FoxO1 phosphorylation, proving that FoxO1 was regulated by exendin-4 via the AMPK pathway.

Clinical trials have demonstrated the protective effect of GLP-1RA on cardiovascular function in type 2 diabetes. However, the mechanism of GLP-1RA-prevented cardiovascular dysfunction is complex and remains unclear. Here, we explored a novel view that exendin-4 increased endothelial hSR-BI/CLA-1 to activate eNOS via the AMPK/FoxO1 pathway in vitro. However, in vivo and clinical studies are required to confirm our findings through further investigation. 

## 5. Conclusions

In this study, we demonstrated that GLP-1RA and exendin-4 upregulated the expression and transcription of hSR-BI/CLA-1 via the AMPK/FoxO1 pathway and consequently induce the activation of eNOS in HUVECs, which may contribute to understanding the mechanism of GLP-1RA’s effect on protecting endothelial function.

## Data Availability

The data presented in this study are available on request from the corresponding author.
